# The OptoReg system: a simple and inexpensive solution for regulating water oxygen

**DOI:** 10.1093/conphys/coae024

**Published:** 2024-05-11

**Authors:** Rasmus Ern, Fredrik Jutfelt

**Affiliations:** Department of Biology, Norwegian University of Science and Technology, Høgskoleringen 5, 7034 Trondheim, Norway; Department of Biology, Norwegian University of Science and Technology, Høgskoleringen 5, 7034 Trondheim, Norway; Department of Biology and Environmental Sciences, University of Gothenburg, Medicinaregatan 7B, 413 90 Gothenburg, Sweden

**Keywords:** Cleware GmbH, FireSting-O2 meter, hyperoxia, hypoxia, kilopascal (kPa), oxygen partial pressure (PO2), oxygen regulator, percent air saturation (% AS), PyroScience, RasmOx

## Abstract

This paper describes an optocoupler-based regulation apparatus for saturation manipulation of oxygen in water (OptoReg). This system enables control of solenoid valves for oxygen and nitrogen gases using a FireSting-O_2_ meter, an optocoupler box and an electronic switch box. The hardware components connect to a computer through Universal Serial Bus (USB) cables. The control software is free and has a graphical user interface, making it easy to use. With the OptoReg system, any lab with a computer running Microsoft Windows operating system and a 4-channel FireSting-O_2_ meter can easily and cheaply set up four independently controlled systems for regulating water oxygen levels. Here, we describe how to assemble and run the OptoReg system and present a data set demonstrating the high precision and stability of the OptoReg system during static acclimation experiments and dynamic warming trials.

## Teaser

This paper introduces a new system for regulating water oxygen levels, using the FireSting-O2 meter. This OptoReg system is inexpensive, easy to build and user-friendly. This paper provides detailed instructions on building and using the system, and it presents a dataset that illustrates the system’s accuracy and stability.

## Introduction

Water oxygen is, like water temperature (Brett, 1971), an ‘abiotic master factor’ because of its effects on aquatic organisms (Vaquer-Sunyer and Duarte, 2008; Chu and Tunnicliffe, 2015; Ern *et al.,* 2016; Ern, 2019; Woods *et al.,* 2022; Ern *et al.,* 2023). Climate change and eutrophication are expanding the oxygen minimum zones (OMZs) in the world’s ocean and increasing the number of aquatic oxygen deficient (hypoxic) “dead zones” in costal and estuarine areas (Diaz and Rosenberg, 2008; Rabalais *et al.,* 2010; Breitburg *et al.,* 2018). Additionally, hyperoxic water can occur as a result of aquatic warming and high levels of photosynthetic oxygen production by marine plants and algae, but the potential effects on aquatic organisms are understudied (McArley *et al*., 2020). Investigating the effects of deoxygenation and hyperoxygenation on aquatic organisms is essential for understanding the important role of oxygen, as well as the impacts of climate change, on aquatic ecosystems. Consequently, the impact of water oxygen on aquatic organisms is an increasingly important research topic (IPCC, 2022).

**Figure 1:**
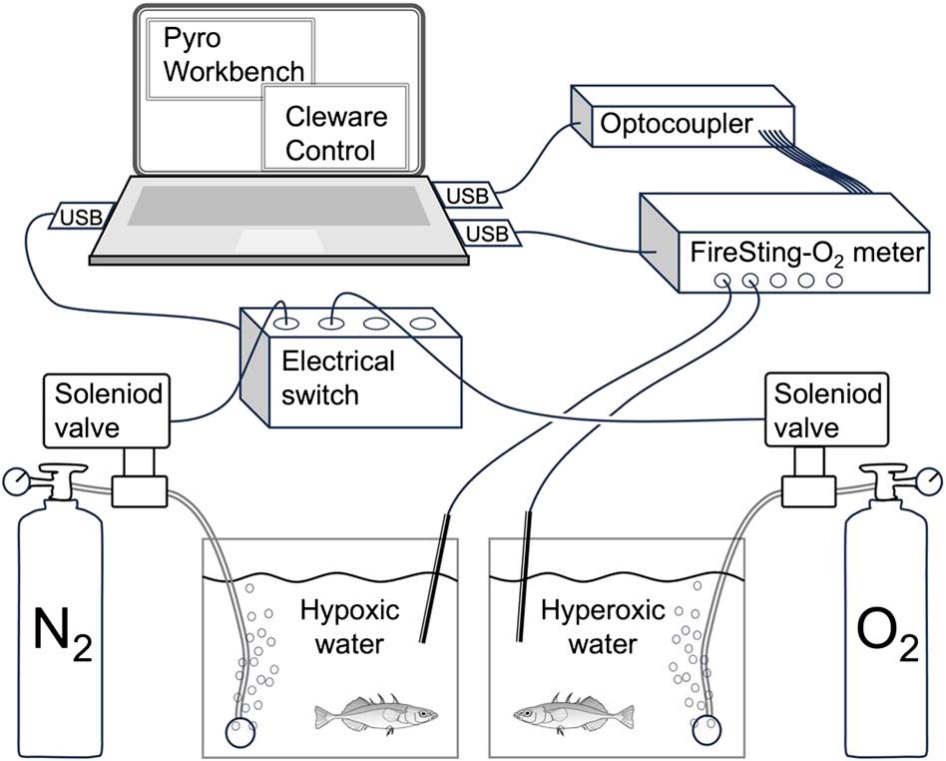
A schematic drawing of the OptoReg system, illustrating its function in regulating water oxygen levels in two tanks: one with hypoxic water and one with hyperoxic water. The FireSting-O_2_ oxygen meter measures the water oxygen level using fibre-optic oxygen sensors. If the water oxygen level exceeds or falls below preset threshold values, which can be configured in the Pyro Workbench software (see text in Box 1), a signal is transmitted from the oxygen meter to the optocoupler. The optocoupler relays the signal to the computer, where it is registered by the ClewareControl software (see text in Box 2). The ClewareControl software controls an electrical switch connected to a solenoid valve. This switch turns ON when the signal from the oxygen meter is present (i.e. when water oxygen levels exceed or fall below threshold values). Turning ON the switch opens the solenoid valve, initiating bubbling with nitrogen and oxygen, respectively.

**Table 1:** Hardware specifications and purchasing details (at the time of publication) on the equipment used to build an OptoReg system and on FireSting-O_2_ systems

Item	Stock No	Price (€)	Quantity
Cleware-shop.de			
USB Optocoupler^a^ (OptoIn)	54	60	1
USB Switch 4	11–3	155	1
RS-online.com			
PCB terminal block	712–4487	20	5
Wago terminal block	883–7557	10	10
Equipment wire	361–579	28	100 m
Solenoid valve (for nitrogen)	307–0248	58	1
Power cable	490–245	9	1
Push-in fitting	212–9173	15	10
Tubing	144–7829	123	150 m
Y tube-to-tube adaptor	916–0918	22	10
Pyroscience.com			
FireSting-O_2_ (1 Channel)	FSO2-C1	2.280	1
FireSting-O_2_ (4 Channels)	FSO2-C4	4.480	1

a ^a^When ordering the Universal Serial Bus (USB) Optocoupler from Cleware, add a note in the remarks field that you need 2500-mV input. Then, they will adapt the resistance for this setting.

The physiology of fishes and other water-breathing ectotherms can be affected by water oxygen through changes in oxygen availability (Farrell and Richards, 2009). Identifying traits that describe the physiological responses of aquatic organisms to water oxygen and determining the oxygen levels at which these traits either manifest or change can pinpoint threshold values beyond which aquatic hypoxia may begin affecting animals at both individual and population levels. Such knowledge can contribute to conservation efforts by improving species distribution models that are parameterized with physiological data, as such mechanistic models are suggested to be more robust in forecasting distribution changes under future environmental conditions (Evans *et al.,* 2015).

Research on the effects of water oxygen levels on aquatic organisms has been partially hampered by the limited availability of high-precision systems for controlling water oxygen in laboratory settings. Commercial systems cost tens of thousands of euros, putting them out of reach for many researchers with limited funding. Do-it-yourself (DIY) systems based on Arduino and Raspberry Pi platforms have been developed and are cheaper, but require both engineering and programming skills, which can also put them out of reach for many researchers. The ability to regulate water oxygen levels in laboratory settings has therefore required either large funds for available commercial systems or the technical skills to build and program custom systems. To address this issue, we have designed a system for regulating water oxygen levels using the common FireSting-O_2_ oxygen meter in combination with commercially available optocouplers, electrical switches, solenoid valves and software with a graphical user interface. Below, we describe how to assemble and run this optocoupler-based regulation apparatus for manipulating oxygen saturation in water (OptoReg), and we present a data set demonstrating precise oxygen regulation during a static acclimation experiment and during a dynamic warming trial.

## Materials and Methods

The OptoReg system uses an optocoupler to relay an electrical signal from an oxygen meter to an electrical switch controlling a solenoid valve. When the water oxygen level reaches a predetermined threshold value, the solenoid valve opens and releases a gas (nitrogen for hypoxia experiments and oxygen for hyperoxia experiments) into the water (Fig. 1).

A solenoid valve is an electromechanical device that controls the flow of gas (or liquid). In the OptoReg system, solenoid valves control the bubbling of nitrogen or oxygen gas. Nitrogen bubbling reduces the water oxygen level and oxygen bubbling increases the water oxygen level. A solenoid valve has an inlet port marked with a P (Pressure) and an outlet port marked with an A (Air/Atmosphere). In a normally closed valve, the type used in the OptoReg system, the valve is closed when no electric current is applied and opened by applying an electric current. Nitrogen is an inert gas and can, therefore, be controlled using most types of solenoid valves. By contrast, oxygen gas supports combustion and MUST ONLY be controlled using solenoid valves that are approved for use with oxygen. Solenoid valves that are not approved for oxygen can create electrical sparks when turned on. These sparks can ignite, potentially causing explosive fire!

**Figure 2:**
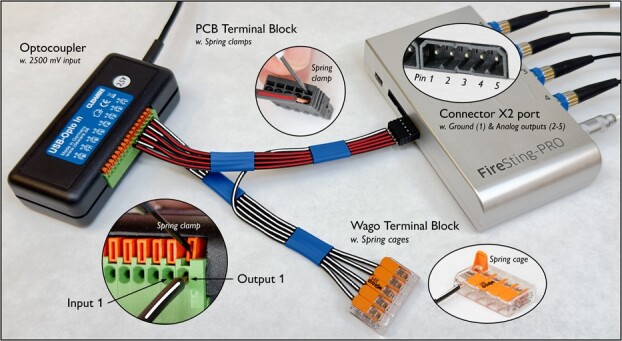
How to connect the analog outputs on the FireSting-O_2_ to the inputs on the Optocoupler, and outputs on the Optocoupler to the ground on the FireSting-O_2_. The four analog outputs (Pin 2–5) in the Connector X2 extension port on the FireSting are connected to the four inputs (1–4) of the first four contacts on the Optocoupler, using a PCB Terminal Block (red/dark lined wires). The four outputs (1–4) of the first four contacts on the Optocoupler are all connected to the ground (Pin 1) in the Connector X2 extension port on the FireSting, using a Wago Terminal Block (white/light lined wires). The last four contacts (5–8) on the Optocoupler are not used. Wires are attached to the Optocoupler and the PCB Terminal Block using Spring Clamps and to the Wago Terminal Block using Spring Cages. The spring clamps are pushed opened using the tip of a 2-mm flathead screwdriver.

An optocoupler is an electronic component that uses light to transmit electrical signals between two electrical circuits—an input circuit and an output circuit—while keeping them electrically isolated from each other. An optocoupler consists of a light-emitting diode (LED) that is connected to the input circuit and a phototransistor that is connected to the output circuit. The LED emits light when a voltage from the input circuit is applied to it. The phototransistor detects the light and generates a current proportional to the light intensity. Keeping the two circuits electrically isolated during signal transmission prevents interference, which would otherwise cause errors in data transmission. An optocoupler is therefore used in the OptoReg system to transmit signals between the low-voltage FireSting-O_2_ system and the high-voltage switch into which the solenoid valves are connected.

The OptoReg system described below is built using oxygen meters, sensors and control software from PyroScience (pyroscience.com); optocouplers, electrical switches and control software from Cleware (cleware-shop.de); and solenoid valves from Burkert (burkert.com). All components are commercially available and affordable (Table 1).

### Hardware

The FireSting-O_2_ Oxygen meter from PyroScience comes with up to four channels for fibre-optic oxygen sensors, one temperature sensor and a USB cable for connecting it to a computer (see the official manual for how to operate the FireSting oxygen meter). The back of the FireSting oxygen meter has an integrated Connector X2 extension port with one ground (Pin 1) and four analog outputs (Pin 2–5) (Fig. 2). The four analog outputs have a range of 0–2500 mV. The 8-channel USB Optocoupler from Cleware has eight contacts (1–8) and a USB cable for connecting it to a computer. The contacts go from right to left, with Contact 1 being furthest to the right and Contact 8 being furthest to the left. Each contact has an input (positive, left clamp) and output (negative, right clamp) (Fig. 2). A contact is open when no voltage (0 mV) is applied to the input and closed when voltage (2500 mV) is applied to the input. When ordering the USB Optocoupler from Cleware, add a note in the remarks field that you need 2500-mV input. The 4-channel USB-Switch from Cleware has four electrical switches (Socket 1–4), a USB cable for connecting it to a computer and an electrical cord with a wall plug. The OptoReg system requires a computer with a minimum of three USB ports, but it will also function using a 3-port USB hub.

The FireSting-O_2_ Oxygen meter is connected to the Cleware Optocoupler using a PCB Terminal Block, a Wago Terminal Block and equipment wires (Fig. 2). The signal from the Optocoupler is relayed to the Cleware USB-Switch using the ClewareControl software as detailed below (Box 2).

### Software

Pyro Workbench software is used to control the four analog outputs on the FireSting-O_2_ meter via the ‘Alarm if out of

**Figure:**
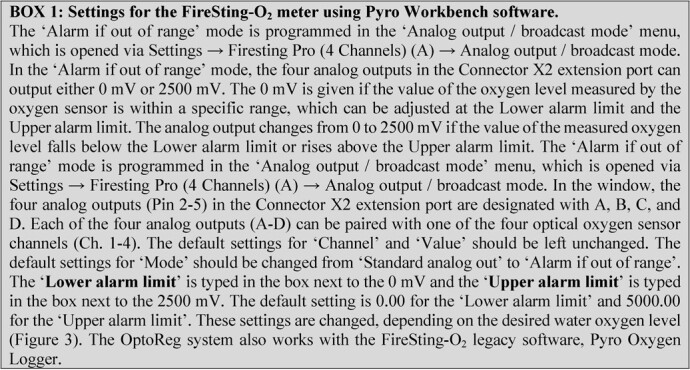


**Figure:**
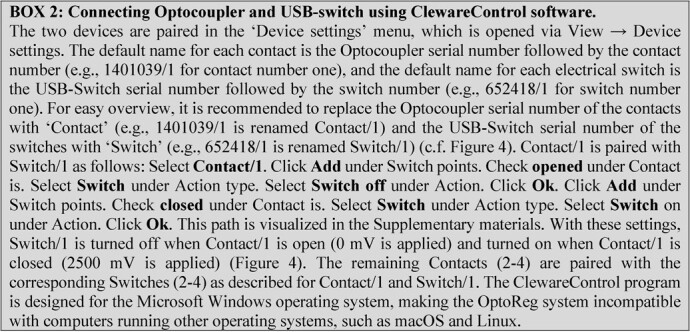


range’ mode ( ). In this mode, the outputs can be set to change from 0 to 2500 mV if the water oxygen level falls below a Lower alarm limit or rises above an Upper alarm limit (Fig. 3). When the voltage of an output changes from 0 to 2500 mV, the connected contact on the Optocoupler (c.f. Fig. 2) is opened. The ClewareControl software is used to pair the first four contacts on the Optocoupler with the four electrical switches on the USB-Switch, in a manner that turns a switch ON when the paired contact is opened and turns in OFF when the contact is closed (Box 2, Fig. 4). Turning on a switch will activate the connected solenoid valve, which will bubble the water with either nitrogen or oxygen, depending on the desired water oxygen level (as detailed below).

**Figure 3:**
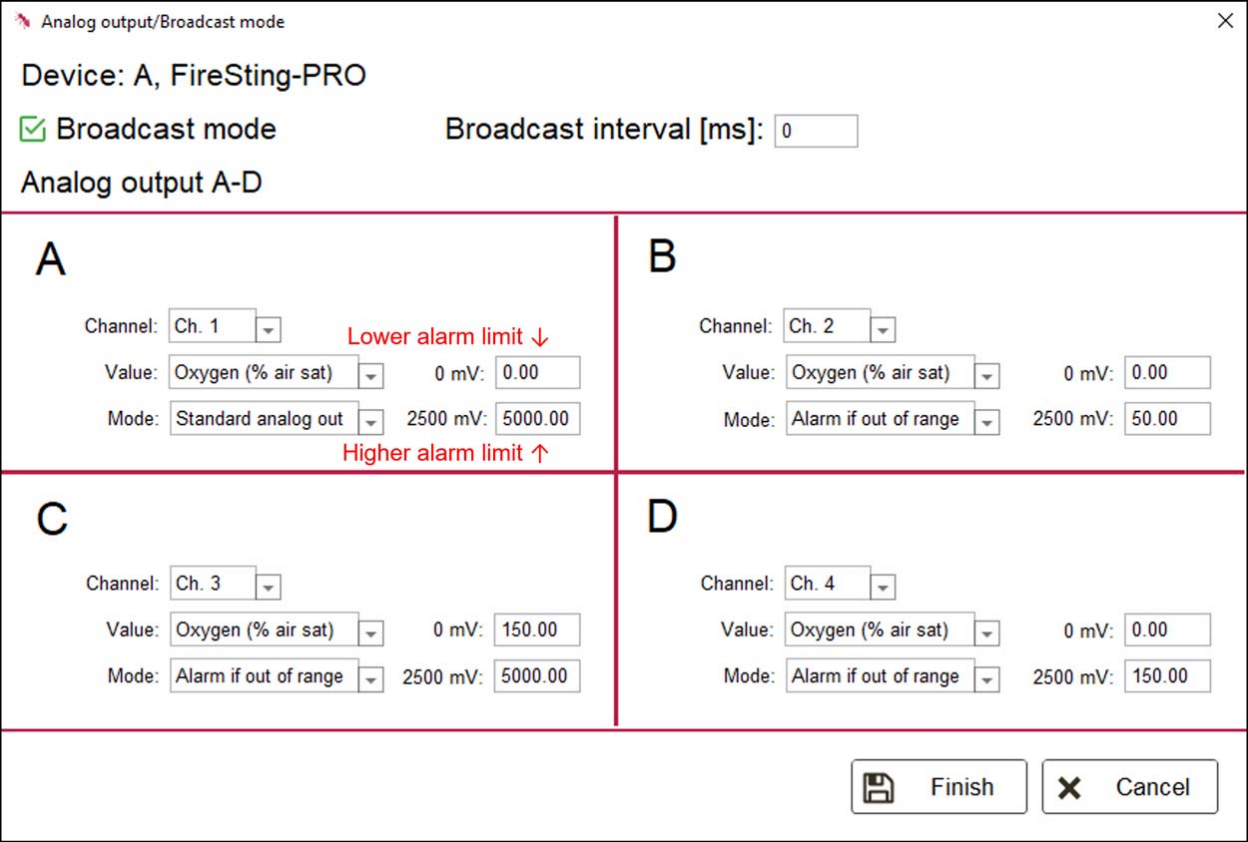
Settings for regulating water oxygen using the ‘Alarm if out of range’ mode in the Pyro Workbench control software for the FireSting-O_2_ meter. The Lower alarm limit and the Higher alarm limit are indicated for the Analog output A. Output **A** shows default settings; output **B** shows settings for maintaining the water oxygen level at 50% air saturation using solenoid-controlled nitrogen bubbling; output **C** shows settings for maintaining the water oxygen level at 150% air saturation using solenoid-controlled oxygen bubbling, and output **D** shows settings for maintaining water oxygen level at 150% air saturation using passive oxygen bubbling and solenoid-controlled nitrogen bubbling. See text and Box 1 for details.

**Figure 4:**
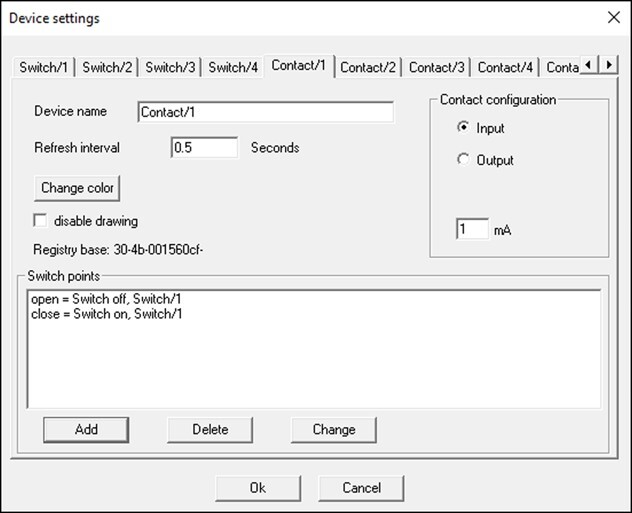
Settings for turning Switch/1 off when Contact/1 is open (0 mV is applied) and for turning Switch/1 on when Contact/1 is closed (2500 mV is applied). See test, Box 2 and Supplementary materials for details.

### Regulating water hypoxia (v1) using solenoid-controlled nitrogen bubbling

In the Pyro Workbench software, the Lower alarm level is set to 0% of air saturation and the Upper alarm level is set to the desired water oxygen level (e.g. 50% air saturation) (Fig. 3, Output A). With this setting, the voltage from the analog output will be 0 mV when the water oxygen level is between 0% air saturation and 50% air saturation, and it will be 2500 mV when the water oxygen level is above.

50% air saturation (or below 0% air saturation). When activated, and starting with normoxic water, this setting will bubble nitrogen gas into the water until the water oxygen level declines below 50% air saturation, at which point the solenoid valve is closed. Whenever the water oxygen level rises above 50% air saturation, because of the passive diffusion of oxygen into the water, the solenoid valve is opened, and the water is bubbled with nitrogen until water oxygen level again declines below 50% air saturation.

### Regulating water hypoxia (v2) using a water pump with aerated water

In tanks where the animal volume is sufficiently large such that their rate of oxygen consumption exceeds the diffusion rate of oxygen into the water, hypoxia can occur. In such cases, hypoxia can be regulated by replacing the solenoid valve with a water pump connected to a tank with aerated water. In the Workbench software, the Lower alarm level is set to the desired water oxygen level (e.g. 50% air sat) and the Upper alarm level is set to 5000% air saturation. With this setting, the voltage from the analog output will be 0 mV when the water oxygen level is between 50% air saturation and 5000% air saturation, and it will be 2500 mV when the water oxygen level is below 50% air saturation. Whenever the water oxygen level drops below 50% air saturation, because of the continuous consumption of oxygen by the animal, the water pump is turned on, and the aerated water is pumped into the tank until the water oxygen level again increases above 50% air saturation. This solution regulates water hypoxia without solenoid-controlled nitrogen bubbling, making it well suited for long-term hypoxia acclimation experiments. One potential confound using this method is that water chemistry can be affected because of accumulation of carbon dioxide and nitrogen waste from the animals.

### Regulating water hyperoxia (v1) using solenoid-controlled oxygen bubbling

When using pure oxygen gas, the solenoid valve must be suitable for use with oxygen. In the Workbench software, the Lower alarm level is set to the desired water oxygen level (e.g. 150% air sat) and the Upper alarm level is set to 5000% air saturation (Fig. 3, Output C). With this setting, the voltage from the analog output will be 0 mV when the water oxygen level is between 150% air saturation and 5000% air saturation, and it will be 2500 mV when the water oxygen level is below 150% air saturation. When activated, and starting with normoxic water, this setting will bubble oxygen gas into the water until the water oxygen level rises above 150% air saturation, at which point the solenoid valve is closed. Whenever the water oxygen level decreases below 150% air saturation, for example, from diffusion of oxygen out of the water, the solenoid valve is opened, and the water is bubbled with oxygen until water oxygen level again rises above 150% air saturation.

### Regulating water hyperoxia (v2) using continuous oxygen bubbling and solenoid-controlled nitrogen bubbling

Hyperoxia can alternatively be controlled using continuous oxygen bubbling instead of solenoid-controlled oxygen bubbling. In this setup, the rate of oxygen bubbling is manually adjusted to a fixed rate by regulating the outgoing pressure on the regulator. In the Workbench software, the Lower alarm level should be set to 0% air saturation and the Upper alarm level should be set to the desired water oxygen level (e.g. 150% air sat) (Fig. 3, Output D). With this setting, the voltage from the analog output will be 0 mV when the water oxygen level is between 0% air saturation and 150% air saturation, and it will be 2500 mV when the water oxygen level is above 150% air saturation. When activated, and starting with normoxic water, the continuous oxygen bubbling will raise the water’s oxygen level until the oxygen level rises above 150% air saturation. When this happen, the solenoid valve controlling the flow of nitrogen gas is opened, and the water is bubbled with nitrogen until the oxygen level decreases below 150% air saturation, at which point the valve is closed and the water oxygen begins to increase because of the continuous oxygen bubbling. The rate of nitrogen bubbling should be large enough that the water oxygen level decreases when the solenoid valve controlling the flow of nitrogen gas is opened, despite the continuous oxygen bubbling. This setup allows the regulation of hyperoxic water without requiring a solenoid valve suitable for use with oxygen, which is considered less safe than nitrogen due to its combustible nature.

**Figure:**
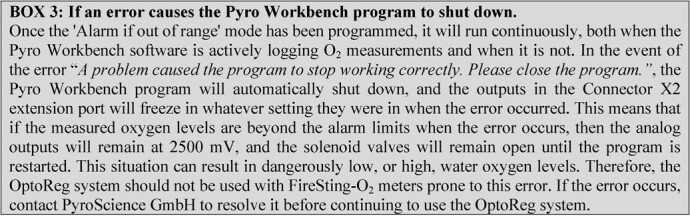


## Results

**Figure 5:**
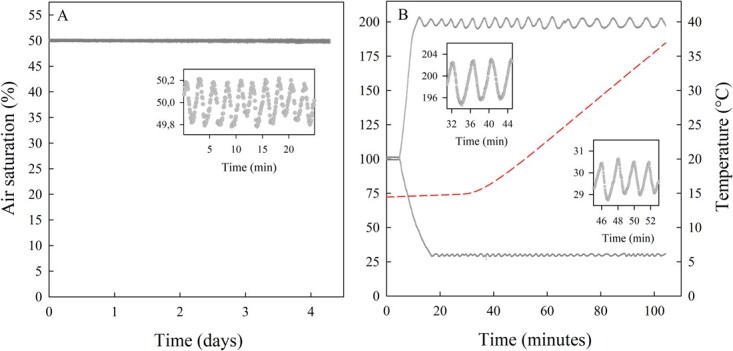
Recorded water oxygen levels during (**A**) a static acclimation experiment (5 days at 20°C) and (**B**) two dynamic warming trials (0.3°C per minute). Individual O_2_ recordings (air saturation, %) are shown with circles. Embedded graphs display zoomed-in inset of O_2_ recordings. Water temperature (°C) during the warming trials is shown with a dashed line (B).

The accuracy and stability of the OptoReg system were tested during a static temperature acclimation experiment and two dynamic warming trials (Fig. 5). The trials were conducted using zebrafish (*Danio rerio*). The acclimation experiment was conducted over a 5-day period, using a 37.5-L tank with a 520 ml per min flow-through of aerated water. The tank contained 40 fish with a mean (±1 SD) body mass of 0.59 ± 0.18 g and a total combined body mass of 23.48 g. The warming trials were conducted using 9.9-L tanks without flow-through water. The tank used in the hyperoxia warming trial contained six fish with a mean (±1 SD) body mass of 0.17 ± 0.09 g and a total combined body mass of 0.99 g, while the tank used in the hypoxia warming trial contained seven zebrafish with a mean (±1 SD) body mass of 0.17 ± 0.06 g and a total combined body mass of 1.20 g. Water mixing during trials was achieved using Eheim universal 300 water pumps (eheim.com). Water temperature was regulated using a SmartPID CUBE—Smart Thermostat application (smartpid.com) and Aqua Medic 500-W titanium heating elements (aqua-medic.de).

The static acclimation experiment was conducted to assess the OptoReg system’s ability to maintain stable oxygen levels over prolonged periods. At the start of the experiment, the system was set to maintain the water oxygen level in the acclimation tank at 50% air saturation using solenoid-controlled nitrogen bubbling (c.f. Fig. 3B). A second, separate, Firesting system was used to independently record oxygen levels. The results showed that the water oxygen level (mean ± 1 SD) during the 5-day acclimation period was maintained at 50.0 ± 0.1% air saturation (Fig. 5A).

The warming trials were conducted to assess the OptoReg system’s ability to simultaneously maintain distinct oxygen levels in different tanks (Fig. 1) and to sustain oxygen levels during fluctuations in water temperature and oxygen solubility. The ramping rate was 0.3°C per minute, which is commonly used to assess the acute upper thermal tolerance of aquatic organisms (Becker and Genoway, 1979; Morgan *et al.,* 2018).

At the start of the warming trials, the OptoReg system was set to maintain one trial tank at 30% air saturation using solenoid-controlled nitrogen bubbling (see Fig. 3B) and to maintain another trial tank at 200% air saturation using solenoid-controlled oxygen bubbling (see Fig. 3C). The results showed that the water oxygen level (mean ± 1 SD) during the warming trials was 198.8 ± 2.4% air saturation in the hyperoxia tank and 29.9 ± 0.4% air saturation in the hypoxia tank (Fig. 5B).

## Discussion

Using a 4-channel FireSting-O_2_ meter, an OptoReg system can regulate water oxygen levels in up to four different systems. It’s important to note that channels dedicated to regulating water oxygen cannot be used for other activities, such as respirometry (Svendsen *et al.,* 2016). Nevertheless, the system can be configured with one, two or three channels specifically dedicated to regulating water oxygen, while the remaining channels are designated for other purposes.

Since the OptoReg system operates on an ‘Alarm’ function, setting a hysteresis is not possible. As a result, users may experience the solenoid switching on and off at a high frequency if the outgoing gas pressure from the regulator is relatively low, potentially causing wear on the solenoid. Increasing the outgoing gas pressure by manually adjusting the regulator will alleviate this issue. When the solenoid valve is opened, the increased bubbling intensity will result in a larger change in the water oxygen level and, consequently, a longer duration until it reaches the threshold value again. When regulating water hypoxia using a water pump with aerated water, instead of solenoid-controlled nitrogen bubbling, using a larger pump will serve the same function as increasing the outgoing gas pressure from the regulator. This type of ‘manual’ hysteresis can be used to reduce the frequency of solenoid switching, but it comes at the cost of increased variance in oxygen levels.

The OptoReg system offers a simple and inexpensive solution for regulating water oxygen using the FireSting-O_2_ meter and sensors. Assembling and operating the system requires neither engineering nor programming skills. The system effectively maintains stable water oxygen levels over extended periods and during acute changes in water temperature. By enhancing the capabilities of laboratories to manipulate water oxygen levels, the OptoReg system is poised to facilitate an increase in studies on the responses of aquatic organisms to changes in water oxygen levels. Consequently, this will contribute valuable data to the field of conservation physiology.

## Data Availability

The data underlying this article will be shared on request to R.E.
